# Metabolic and electrolyte abnormalities as risk factors in drug-induced long QT syndrome

**DOI:** 10.1007/s12551-022-00929-7

**Published:** 2022-01-27

**Authors:** Clifford TeBay, Adam P. Hill, Monique J. Windley

**Affiliations:** 1 Victor Chang Cardiac Research Institute, 405, Liverpool street, Darlinghurst, Sydney, NSW 2020 Australia; 2 St. Vincent’s Clinical School, UNSW Sydney, Sydney, Australia

**Keywords:** Arrhythmia, hERG, Hypokalaemia, Hypomagnesemia, Febrile, Acidosis

## Abstract

Drug-induced long QT syndrome (diLQTS) is the phenomenon by which the administration of drugs causes prolongation of cardiac repolarisation and leads to an increased risk of the ventricular tachycardia known as torsades de pointes (TdP). In most cases of diLQTS, the primary molecular target is the human ether-à-go-go-related gene protein (hERG) potassium channel, which carries the rapid delayed rectifier current (I_Kr_) in the heart. However, the proarrhythmic risk associated with drugs that block hERG can be modified in patients by a range of environmental- and disease-related factors, such as febrile temperatures, alterations in pH, dyselectrolytaemias such as hypokalaemia and hypomagnesemia and coadministration with other drugs. In this review, we will discuss the clinical occurrence of drug-induced LQTS in the context of these modifying factors as well as the mechanisms by which they contribute to altered hERG potency and proarrhythmic risk.

## Drug-induced long QT syndrome

Drug-induced (diLQTS) or acquired long QT syndrome (aLQTS) is characterised by prolongation of the QT interval on the surface electrocardiogram (ECG) and is associated with a markedly increased risk of the potentially lethal ventricular arrhythmia known as torsades de pointes (TdP (Roden 2004)). A prospective study of hospital admission for drug-induced TdP reported 3.3 cases per million over the 4-week study period, translating to an annual incidence of 4/100,000 (Darpö 2001). However, this may be an underestimate for the broader population since TdP is often not reported in out-of-hospital cases (Birda et al. 2018; Lin et al. 2020; Yu et al. 2017). For hospitalised patients, the prevalence of severe diLQTS has been reported as between 1.6 and 3.3% of patients (Birda et al. 2018; Lin et al. 2020; Yu et al. 2017), with these patients having a higher all-cause mortality than their non-LQTS counterparts (Lin et al. 2020; Yu et al. 2017). Over the past 30 years, a range of cardiac (Kannankeril et al. 2011; Selzer and Wray 1964; Singh et al. 2000) and noncardiac (Schoonmaker et al. 1966) drugs have been shown to prolong the QT interval, with several being recalled from the market (Roden 2004). diLQTS can be caused by drugs that block any of the ion channel currents that contribute to normal cardiac repolarisation. In practice, however, the majority of drugs that cause diLQTS do so by inhibiting hERG/Kv11.1 potassium channels, encoded by the *KCNH2* gene, which carries the rapid delayed rectifier K^+^ current (I_Kr_) in the heart (Vandenberg et al. 2012). This unintentional block of hERG is therefore a problem both for development of new therapeutic compounds, as well as management of patients prescribed such drugs (see Table 1 for a full list of compounds discussed in this review). Consequently, screening for potency of hERG channel block, as a surrogate for QT prolongation and repolarisation delay, is a mandated part of preclinical drug development ((ICH S7B 2005), Fig. 1). However, the link between a drug’s potency to block hERG and the emergence of arrhythmia is complex. Of the majority of new chemical entities, up to 70% in some estimates (Shah 2005) can block hERG at some concentration, yet only a small percentage cause arrhythmia (Darpö 2001, 2007). Moreover, even for drugs that are demonstrably “high risk”, the severity of adverse events across the patient population can be highly variable ranging from minimal prolongation of cardiac repolarisation to the induction of lethal arrhythmia (Kannankeril et al. 2011; Singh et al. 2000). A number of factors likely contribute to this variable response, including pre-existing disease resulting in electrical or structural remodelling of the myocardium, sex differences and an individual’s genetic background (Echt et al. 1991; Makkar et al. 1993; Roden and Viswanathan 2005). Aside from these patient-specific factors, a drug’s proarrhythmic propensity can also be modified by other systemic/acquired factors in patients such as electrolyte disturbances, acidosis, febrile temperatures and coadministration with other drugs. The importance of such considerations has been highlighted recently in relation to repurposing of drugs for treatment of COVID-19. Specifically, various combinations of drugs that are known to carry some degree of proarrhythmic risk, including chloroquine, hydroxychloroquine, azithromycin, erythromycin and lopinavir/ritanavir, have been proposed as potential therapies (Delaunois et al. 2021; Zequn et al. 2021) in COVID-19 patients where fever (Aslam et al. 2021; Pan et al. 2020; Zhou et al. 2020), acidosis (Zhou et al. 2020) and electrolyte disturbances (Alfano et al. 2021; Lippi et al. 2020; Stevens et al. 2021) were also reported. Here, we will review both the clinical occurrence of diLQTS in the context of fever, hypokalaemia, hypomagnesemia and other electrolyte disturbances and the mechanisms by which these factors contribute to altered potency of hERG block and proarrhythmic risk.

**Table 1:** Compounds related to TdP development. List of all compounds mentioned in this literature review, their drug class and primary target

Drug name	Drug class	Primary target	Reference
Amiodarone	Class III antiarrhythmic	hERG and CACNA2D2^*^ channel	Du et al. (2011)
Amisulpride	Antipsychotic	Dopamine D2 receptor	Lin et al. (2009)
Astemizole	Antihistamine	Histamine H1 receptor	Yao et al. (2005)
Azimilide	Class III antiarrhythmic	I(Ks) and hERG channels	Busch et al. (1998), Dong et al. (2004)
Azithromycin	Macrolide antibiotic	23 s RNA of the bacterial 50S ribosomal unit	Delaunois et al. (2021), TeBay et al. (2021), Zequn et al. (2021)
Bepridil	Antianginal	L-type calcium channel and Na^+^/K^+^-ATPase pump	Windley et al. (2018)
Berberine	Alkaloid	*Unknown*	Zhi et al. (2015)
Ceftriaxone	Cephalosporin antibiotic	Peptidases of the bacterial cytoplasmic membrane	Lazzerini et al. (2018)
Chloroquine	Antimalarial	Hemozoin	Delaunois et al. (2021), TeBay et al. (2021), Warhurst (1986), Warhurst et al. (2003), Zequn et al. (2021)
Cisapride	Gastroprokinetic	Serotonin 5-HT_4_ receptor	Barrows, et al. (2009), Kamiya et al. (2008), Lacerda et al. (2001), Lee et al. (2019), Lin et al. (2005c), Perrin et al. (2008), Thomas et al. (1998), Thouta et al. (2018), Windley et al. (2016), Windley et al. (2018)
Clarithromycin	Macrolide antibiotic	23 s RNA of the bacterial 50S ribosomal unit	Zhi et al. (2015)
Diltiazem	Antianginal	L-type calcium channel	Thomas et al. (1998)
Disopyramide	Class 1A antiarrhythmic	Fast sodium channels	Hirose et al. (2008), Lazzerini et al. (2018)
Dofetilde	Class III antiarrhythmic	hERG channel	Du et al. 2011, Perrin et al. (2008), Singh et al. (2000), Wang et al. (2016), West et al. (1997), Yang et al. (2004)
Domperidone	Gastroprokinetic	Dopamine D2 and D3 receptor	Boyce et al. (2012)
E-4031	Class III antiarrhythmic	hERG channel	Wang et al. (1997), West et al. (1997), Yao et al. (2005)
Enalapril	Antihypertensive	Angiotensin converting enzyme	Varriale and Ramaprasad (1995)
Encainide	Class 1c antiarrhythmic	Sodium channel protein type 5 subunit alpha	Echt et al. (1991)
Erythromycin	Macrolide antibiotic	23 s RNA of the bacterial 50S ribosomal unit	Delaunois et al. (2021), Guo et al. (2005), Kirsch et al. (2004), Lacerda et al. (2001), Paris et al. (1994)
Fentanyl	Opioid analgesic	µ-opioid receptor	Tschirhart and Zhang (2020)
Flecainide	Class 1C antiarrhythmic	Fast sodium channel	Du et al. (2011), Echt et al. (1991), Paul et al. (2002)
Flupenthixol	Antipsychotic	Dopamine D1 and D2 receptor	Lin et al. (2009)
Gentamicin	Aminoglycoside antibiotic	Lipopolysaccharides and phospholipids and the bacterial cell membrane	Varriale and Ramaprasad (1995)
Glyburide	Sulfonylurea	KATP^**^ channels	Varriale and Ramaprasad (1995)
Halofantrine	Antimalarial	*Unknown*	Charbit et al. (2002)
Haloperidol	Antipsychotic	Dopamine D2 receptor	Lin et al. (2009)
Hydroxychloroquine	Antimalarial	*Unknown*	Delaunois et al. (2021), TeBay et al. (2021), Warhurst et al. (2003), Zequn et al. (2021)
Ibutilide	Class III antiarrhythmic	hERG and slow sodium channel	Lin et al. (2008)
Ibogaine	Psychoactive/psychedelic	µ-, δ- and κ- opioid receptors, serotonin 5-HT_2A_, HT_2C_ and HT_3−_ receptors, sigma σ_1_ and σ_2_ receptors, NMDA*** receptor, nicotinic acetylcholine (nACh) receptor, serotonin transporter (SERT) and dopamine active transporter (DAT)	Thurner et al. (2014)
Itraconazole	Antifungal	14-α-sterol demethylase of the fungal cell membrane	Pohjola-Sintonen et al. (1993)
Ketoconazole	Antifungal	14-α-sterol demethylase of the fungal cell membrane	Boyce et al. (2012) Rajput et al. (2010), Yao et al. (2005)
Lopinavir	Antiretroviral	HIV-1 protease enzyme	Zequn et al. (2021)
Loratadine	Antihistamine	H1 histamine receptor	Lacerda et al. (2001)
Moxifloxacin	Fluoroquinolone antibiotic	Topoisomerase II (DNA gyrase) and topoisomerase IV of the bacteria	Alexandrou et al. (2006)
Posaconazole	Antifungal	14-α-sterol demethylase of the fungal cell membrane	Panos et al. (2016)
Prednisolone	Glucocorticoid	Phospholipase A2	Hirose et al. (2008)
Quinidine	Class I antiarrhythmic	L-type calcium, hERG, slow IKs and KATP** channels	Ayad et al. (2010), Barrows et al. (2009), Dong et al. (2004), Paul et al. (2002), Po et al. (1999), Roden et al. (1986), Selzer and Wray, (1964), Yang et al. (1997)
Quinine	Alkaloid/antimalarial	*Unknown*	Warhurst (1986)
Risperidone	Antipsychotic	Dopaminergic D2 and serotonin 5-HT_2A_ receptors	Lin et al. (2009)
Ritonavir	Antiretroviral	HIV protease inhibitor	Zequn et al. (2021)
Terfenadine	Antihistamine	Histamine H1-receptor	Kamiya et al. (2008), Lacerda et al. (2001), Paris et al. (1994), Perrin et al. (2008), Pohjola-Sintonen et al. (1993), Rajput et al. (2010), Thouta et al. (2018), Windley et al. (2018), Yao et al. (2005)
Thioridazine	Antipsychotic	Dopamine D1 and D2 receptors	Schoonmaker et al. (1966)
Vancomycin	Glycopeptide antibiotic	Peptidoglycan matrix inhibitor of the bacterial cell membrane	Varriale and Ramaprasad (1995)
Verapamil	Class IV antiarrhythmic	L-type calcium channel	Windley et al. (2018), Zhang et al. (1999)

^*^Calcium voltage-gated channel auxiliary subunit alpha2 delta2 gene protein.**ATP-sensitive K^+^ channel.***N-methyl-D-aspartate receptor

**Fig. 1:**
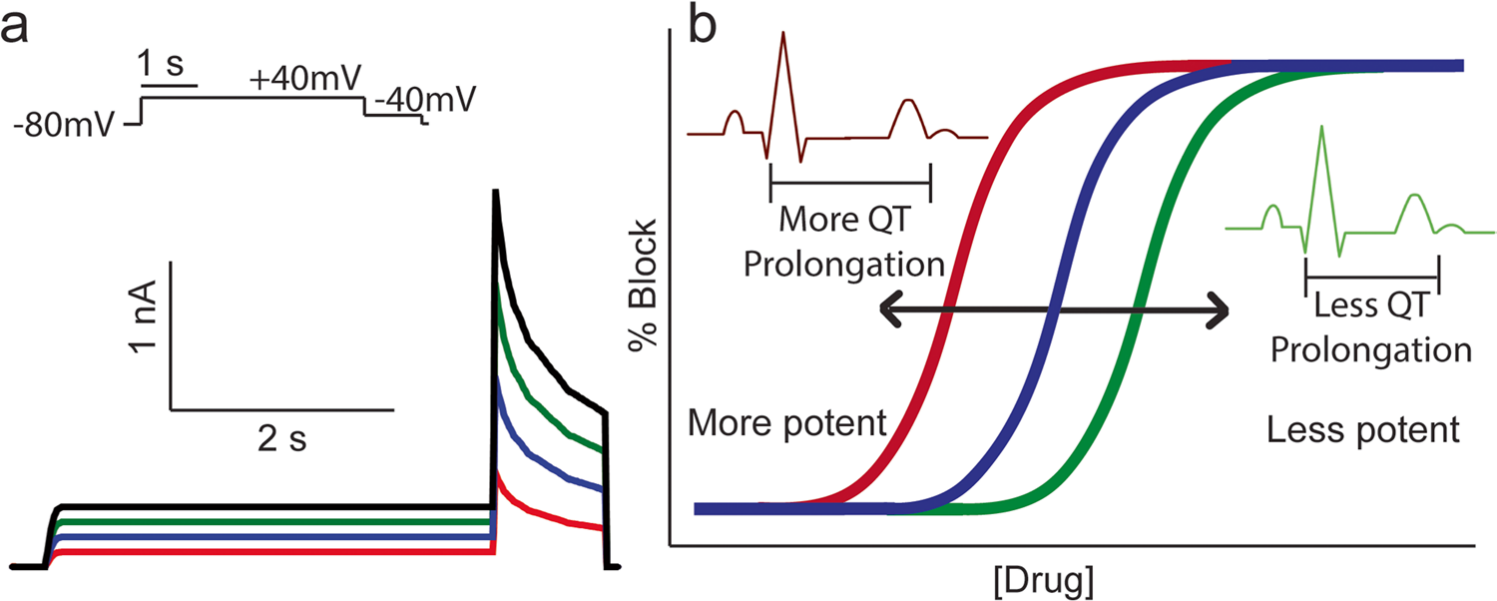
Summary of environmental effects on drug potency. Many disease factors are known to shift the potency of drugs blocking hERG, such as fever, hypokalaemia, hypocalcaemia, etc. **a** A theoretical hERG tail current with scale indicated for current amplitude and time, as elicited by the protocol in the above insert. The black trace represents a control current evoked in drug-free conditions, with the blue trace representing 50% inhibition of the current evoked by a theoretical drug. A condition leading to less potent drug inhibition is represented in green, showing only 25% inhibition, with a condition leading to greater potency leading to 75% inhibition and as depicted in red. **b** A theoretical concentration response curve, with the main drug effect represented in blue. A condition creating lesser potency would lead to a rightward shift, as indicated in green, and, on an ECG, would lead to less QT prolongation, as seen in the insert and depicted in green. Conditions leading to greater potency are depicted in red, and would shift leftward and, on an ECG, would lead to more prolongation, as seen in the insert and as depicted in red. Assets for the ECG traces obtained from Servier Medical Art (Servier 2021)

## Effect of kalaemic variation on drug-induced long QT syndrome

Potassium is the most abundant intracellular cation, which in healthy patients exists within the range of 3.6–5.0 mM in the plasma (El-Sherif and Turitto 2011; Salzman 2018). In the case of altered serum potassium, hypokalaemia is the most common electrolyte abnormality, occurring in over 20% of hospitalised patients, and is defined as a plasma K^+^ level of less than 3.6 mM. This occurs most frequently as a result of decreased intake, increased renal or gastrointestinal loss or via transcellular shift (El-Sherif and Turitto 2011; Salzman 2018). Hyperkalaemia (plasma K^+^  > 5.0 mM) is less common, reported in 8% of hospitalised patients, and occurs as a result of potassium-sparing diuretic use, higher intake, decreased excretion due to renal failure or damage or transcellular shift of potassium into the extracellular environment (El-Sherif and Turitto 2011; Salzman 2018). In patients taking drugs with established proarrhythmic risk, changes in serum K^+^ have been observed to drive further QT prolongation and incidence of TdP. For example, Ayad et al. reported the case of a patient taking quinidine for 15 years without any incidence of QT prolongation who developed TdP and syncope as a result of hypokalaemia by way of gastrointestinal loss (Ayad et al. 2010). Similarly, in a study of 24 individuals, patients administered hERG blockers such as quinidine while taking potassium-depleting diuretics were identified to be at higher risk for QT prolongation and development of TdP, although some of these patients also presented with several other risk factors such as hypertension, cardiomyopathy or were also taking additional QT prolonging drugs (Roden et al. 1986). However, hypokalaemia rarely presents alone, meaning other parallel factors can also contribute to QT prolongation. In a study of 11 patients in whom diLQTS was present, including 8 who exhibited severe hypokalaemia, additional factors such as hypomagnesaemia, hypertension and alcohol use were also present (Digby et al. 2011), while in a larger study of 804 chronic kidney disease patients, lower serum K^+^ and Ca^2+^ were each found to be significant contributors to QT prolongation, often against a background of chronic diseases such as hypertension or diabetes (Liu et al. 2019).

### Mechanism of kalaemia-dependent changes in hERG block and QT prolongation

**Fig. 2:**
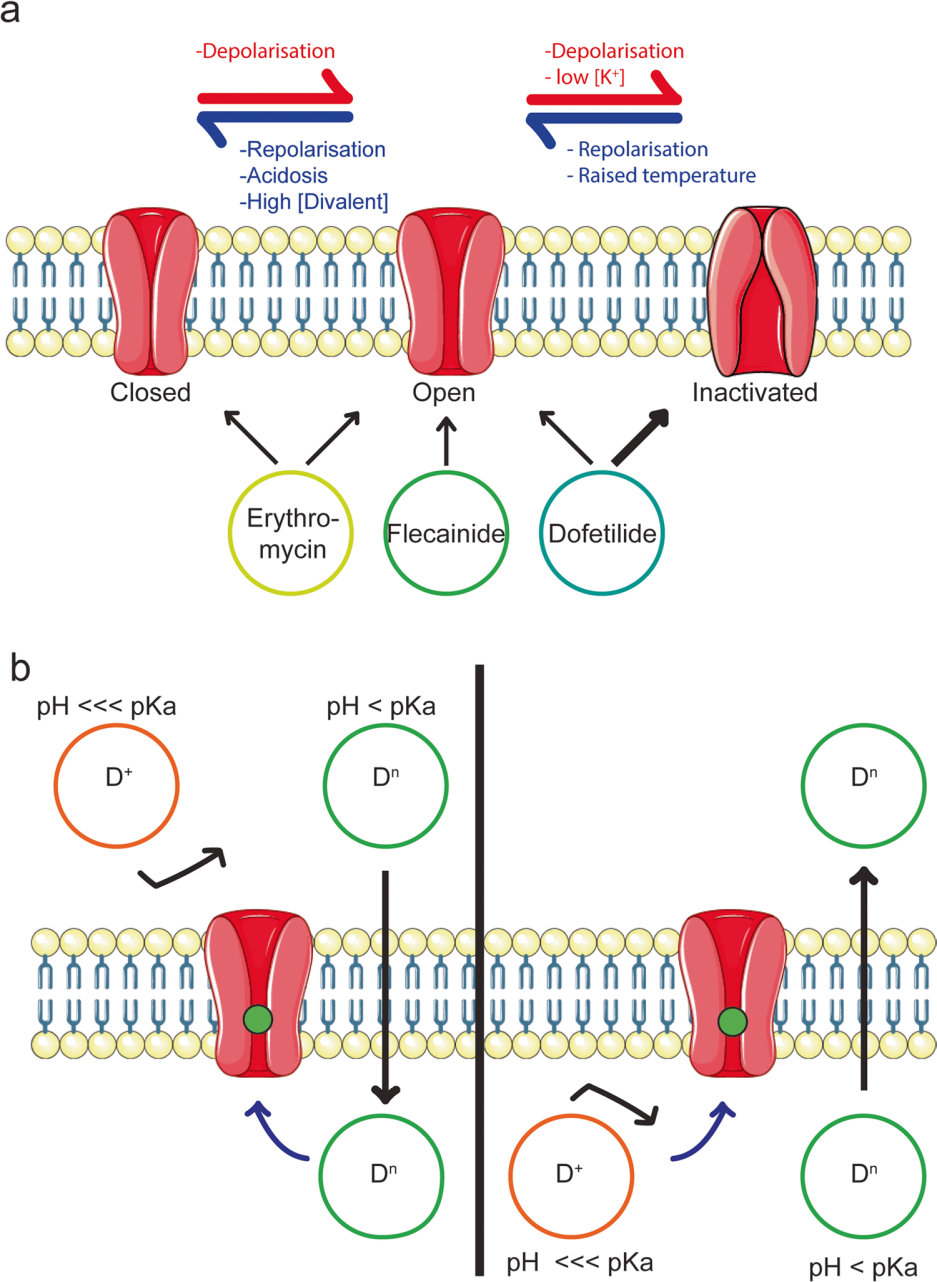
Mechanisms of environmental effects on hERG and drug interactions. Some pathophysiological changes can have effects on the molecular mechanisms of hERG. **a** Represents a schematic showing hERG gating starting in the closed state (left), transitioning through to the open state (middle) by processes of depolarisation and transitioning again to an inactivated state (right) through depolarisation, with the reverse direction of these processes driven by repolarisation. Conditions that can increase deactivation, from open to closed state, include acidosis and high concentration of divalent ions, whereas conditions that could lead to a greater drive to inactivation includes low potassium ion concentration. Finally, raising temperature increases the threshold for hERG to exist in the open state. Beneath are drugs with state preference, with arrows indicated towards which hERG state they possess preferential binding towards, including dofetilide able to bind to open or inactivated state, with greater preference for the latter (Perrin et al. 2008; Wang et al. 2016; Yang et al. 2004), flecainide with open-state preference (Paul et al. 2002) and erythromycin with open or closed state preference (Guo et al. 2005). **b** Indicates the effect of acidosis on drug diffusion across the lipid bilayer. The site of binding is often located such that drug molecules require access from the intracellular side of the membrane and so must be able to cross the cell membrane. The left panel indicates drug administered extracellularly in the presence of extracellular acidosis. Where the local pH is far below the pKa of the drug molecule, a significant proportion of the drug molecule will become charged (D +) and hence unable to cross the lipid bilayer and reach the site of drug binding. Whereas when pH is only slightly below (or above) that of the molecules pKa, a greater proportion is available in the neutral or uncharged state (Dn), which can cross the cell membrane and reach its site of action, indicated by the closed green circle. On the right shows similar conditions yet for intracellular drug application with intracellular acidosis. Here, the difference is that a greater amount of neutral drug molecule would lead to a greater diffusion out of the cell, and hence, less drug is available for channel block, where instead with a local pH far below the drug molecule’s pKa, the drug molecule becomes charged, and hence experiences trapping within the cell, and so a greater amount is available to block the channel. All channels, as well as lipid bilayer assets, were obtained from Servier Mediact Art (Servier 2021)

Understanding the relationship between kalaemic variation and drug-induced prolongation of repolarization is complex, since variation in extracellular potassium has direct effects on cardiac repolarization, via effects on potassium channel function and expression, as well as drug binding (Barrows et al. 2009; Guo et al. 2009, 2011; Limberis et al. 2006; Melgari et al. 2014; West et al. 1997; Yang et al. 2004, 1997). Here we will focus on studies that have specifically addressed potassium dependence of a drug’s potency to block hERG. Across the literature, reports of the influence of K^+^ on potency to block hERG across drugs is broadly consistent, with increasing extracellular potassium reducing the potency of block (Barrows et al. 2009; Busch et al. 1998; Lin et al. 2007, 2008, 2005c; Lin and Papazian 2007; Mergenthaler et al. 2001; TeBay et al. 2021; Wang et al. 1997; West et al. 1997; Yang et al. 2004) and decreased potassium concentration increasing potency of block (Lin et al. 2005a; TeBay et al. 2021; Tschirhart and Zhang 2020). Two potential mechanisms have been proposed to explain this. First, it has been suggested that changes in the state or conformation of hERG as a function of K^+^ might impact the potency of drugs that exhibit state-dependent binding. The hERG channel can exist in one of three states: closed, open or inactivated, with two voltage-dependent gates, a fast inactivation gate and a slow activation/deactivation gate (Vandenberg et al. 2012). Some drugs can exhibit “state preference”, showing a greater affinity for either open or inactivated state (Lee et al. 2016, 2019; Perrin et al. 2008; Stork et al. 2007). As a result, changes in conditions such as K^+^ that is known to alter the equilibrium between the open and inactivated states of the channel can contribute to variation in observed potency for state-dependent drugs. For example, after observing reduced potency across a panel of drugs with inactivated state preference in the presence of elevated K^+^, Yang et al. posited that the shift away from the inactivated state of the hERG channel that occurs under these conditions (Fig. 2a) would reduce the observed degree of block (Yang et al. 2004). Supporting this idea, it has also been shown (in the absence of variation of external K^+^) that hERG mutants with reduced inactivation could greatly attenuate the block of drugs with inactivated state preference such as cisapride and terfenadine (Perrin et al. 2008), while voltage protocols that drive occupancy of the inactivated state result in a higher observed potency for state-dependent drugs (Lee et al. 2016, 2019). However, there is also evidence to counter the concept of state-dependent binding underlying the effect of potassium. Barrows et al. showed that despite significant reduction in hERG potency for cisapride and quinidine with increasing K^+^ between 0 and 20 mM K^+^, there was little change in the fraction of channels existing in inactivated state at + 20 mV between these two potassium concentrations. Based on this evidence, they reasoned that state preference of block did not underpin the altered potency seen for these drugs (Barrows et al. 2009). Similarly, though again outside of a K^+^ context, Thouta et al. used mutants that were constitutively open to explore the preference of terfenadine or cisapride for binding to the open or inactivated state and were able to show that degree of drug block did not change in accordance with the extent of inactivation, suggesting that these two drugs do not exhibit an inactivation state preference (Thouta et al. 2018).

The second mechanism proposed to explain the potassium dependence of a drug’s potency to block hERG is that electrostatic repulsion between the K^+^ ion and the bound drug molecule induces a “knock-off” effect (Barrows et al. 2009; Wang et al. 1997). Wang et al. showed that an inactivation-deficient mutant (S631C, G628C) had near identical external K^+^ sensitivity for E-4031 block as the wild-type channel (Wang et al. 1997) and proposed that since both potassium and E-4031 possess a single positive charge, an electrostatic repulsion mechanism could explain the effect of potassium on drug potency. The study found that with the differences in K^+^ they had used (2 mM vs 98 mM), there would be sufficient free energy to account for the observed reduction in block (Wang et al. 1997). Further to this, it has been proposed that the ability of monovalent cations to “knock off” a drug from its binding site on the hERG channel depends on the ion’s permeability (Barrows et al. 2009). Evidence for this includes a correlation between the observed degree of potency of block for cisapride and quinidine and ionic permeability when the permeant ion or chemical species is switched between potassium, rubidium, caesium and TEA, where the degree of block follows the ion’s permeability through hERG of *P*_K+_  = *P*_Rb+_  > *P*_Cs+_  >  > *P*_TEA_ (Barrows et al. 2009). However, sensitivity of block to specific monovalent ions is also drug dependent, as the degree of block for quinidine was significantly different between 2 and 20 mM K^+^, as well as between K^+^ and Cs^+^, whereas cisapride block was unchanged (Barrows et al. 2009).

In reality, it is likely that both mechanisms may contribute, depending on the specific compound. In the current literature, mechanistic studies have generally sampled only small subsets of drugs, often because data has been generated using manual patch-clamp electrophysiology, which limits the throughput and scale of these investigations. To more confidently discern the mechanism by which altered K^+^ affects drug potency, it is likely that studies of larger drug panels are required, which could be facilitated using high-throughput platforms such as automated patch-clamp or radioligand binding assays. For example, Diaz et al. used ^3^H dofetilide binding assay to assess a panel of 56 compounds, showing that higher K^+^ lead to reduced potency for some compounds, though increased potency for others (Diaz et al. 2004) — inconsistent with the broad trend reported in prior patch-clamp studies. However, in comparison with the gold standard of manual patch clamp, there was a greater than 5- to sixfold difference between potencies measured in binding versus patch clamp for some compounds, with 6 of those compounds having greater than tenfold difference (Diaz et al. 2004). In resolving this question, the use of automated patch-clamp platforms, which combine throughput with gold standard electrophysiology, is likely the technology that will facilitate the scale and quality of information required for interpreting and predicting the clinical implications of K^+^ on hERG drug block and proarrhythmic risk into the future.

## Effects of divalent ions on drug-induced long QT syndrome

### Clinical observations for altered serum divalent concentration

Two divalent cations that are (i) present in human plasma at concentrations relevant for modification of hERG function and/or block, and (ii) have altered concentrations in pathophysiological states, are magnesium and calcium. In healthy patients, normal total plasma calcium concentration is in the range 2.2–2.55 mM, where concentrations outside of this range, typically lower, can contribute to QT prolongation and hence arrhythmic risk (Liu et al. 2019; Nijjer et al. 2010; Szymanski et al. 2013). However, free- or ionised-calcium concentrations are significantly lower (1.05–1.3 mM (Goldberg 2019)), due to binding to plasma proteins such as albumin (Labriola et al. 2009), making this the preferred clinical measurement in predicting prolongation of the QT interval (Kim et al. 2019) and a more suitable comparison for in vitro experiments than total Ca^2 +^ . Hypocalcaemia can be observed with renal insufficiency, parathyroid disease, reduced intake, acute pancreatitis, septic shock or other electrolyte disturbances, whereas hypercalcaemia is associated with hyperparathyroidism, vitamin D disturbances, endocrine disorders, neoplastic disorders and many other malignancies (El-Sherif and Turitto 2011; Salzman 2018). For magnesium, the normal range is 0.7–0.95 mM, and while both hypomagnesemia and hypermagnesemia can result in QT interval prolongation (Topf and Murray 2003), their effects on electrophysiology are often hard to ascertain due to their frequent association with other electrolyte or electrophysiological abnormalities (Ayad et al. 2010; El-Sherif and Turitto 2011; Roden et al. 1986; Salzman 2018; Whang and Ryder 1990). Hypomagnesemia is common, especially in geriatric populations, and can occur due to decreased gastrointestinal uptake or renal loss, whereas hypermagnesemia is far rarer, especially outside of an obstetric population, given the large reserve of magnesium excretion potential the kidneys possess, often only occurring in the background of renal failure (El-Sherif and Turitto 2011; Topf and Murray 2003).

### Mechanism of divalent ion-dependent changes in hERG block

While there is significant literature on the effect of divalent cations on cardiac electrophysiology and hERG channel function, there are fewer comprehensive reports on divalent cation dependence of hERG drug block potency. Furthermore, the literature that does exist presents a somewhat inconsistent narrative. Increased extracellular Mg^2+^ has been shown to increase the potency of hERG block for multiple compounds (Po et al. 1999; TeBay et al. 2021), whereas reduced internal Mg^2+^ was found to reduce the potency of quinidine (Yang et al. 1997). Conversely, concentrations of extracellular Ca^2+^ between 0.1 and 10 mM did not modify the block of either quinidine or cisapride (Barrows et al. 2009). Since there are suggestions that divalent ions could act as hERG/IKr blockers themselves, with binding sites identified within the hERG channel (Anumonwo et al. 1999; Ho et al. 1996, 1998, 1999), one potential mechanism could be that divalent ions together with hERG blocking drugs could result in an increased overall load of IKr inhibition (Po et al. 1999). Another potential explanation is that divalent ions regulate the deactivation kinetics of hERG, which could in turn affect drug dissociation and the degree to which some drugs exhibit “drug trapping” (Barrows et al. 2009). One factor that has confounded in vitro investigations in this area is the need for 1–2 mM concentrations of calcium in bath solutions for patch-clamp electrophysiology, which is critical for formation and maintenance of high-quality seals (Lin and Papazian 2007). As a result, investigations of the effects of variation in divalent ion concentrations in the physiological range are limited in these systems. This issue is particularly salient in automated high-throughput patch-clamp systems, where calcium fluoride seal enhancers are critical in establishing high-quality seals (Braun et al. 2021), meaning thorough investigation of the effects of divalent ions on drug block of hERG at large scale remains technically difficult. In addition to this practical challenge, there is also the issue of what is physiologically or clinically relevant. While observing the effects of wide ranges in concentration of divalent ions may be mechanistically interesting, calcium and particularly magnesium exist in narrow physiological ranges, meaning the clinical relevance of such studies are limited.

## Acidosis and alkalosis

### Effect of acidosis and alkalosis on drug-induced QT prolongation

Metabolic acidosis can increase the QT interval on the ECG (Yenigun et al. 2016) as well as lower the threshold for ventricular fibrillation. Such changes can become particularly problematic in the case of localised changes in pH surrounding ischemic regions of the heart, which produce heterogeneity in action potential duration and provide an electrical substrate for re-entry (Clayton and Holden 2005; Gebert et al. 1971; Podrid and Myerburg 2005; Surawicz 1985). Of specific relevance to this review, acidosis has also been reported as a comorbidity in cases of diLQTS (Riezzo et al. 2009). In relation to hERG channels, changes in pH can directly affect hERG function (Anumonwo et al. 1999; Jiang et al. 1999; Jo et al. 1999; Lin et al. 2005a; Shi et al. 2014; Van Slyke et al. 2012; Vereecke and Carmeliet 2000) as well as the molecular pharmacology of the drug channel interaction. In the latter case, early experiments showed that a reduction in pH to 6.8 could significantly reduce hERG block by dofetilide (West et al. 1997). Across numerous subsequent reports, there is broad consensus that extracellular acidification reduces hERG block by a range of compounds (Du et al. 2011; Lin et al. 2005a, b, 2008; TeBay et al. 2021; Thurner et al. 2014; Tschirhart and Zhang 2020; Wang et al. 2016; Zhang et al. 1999), with alkalisation enhancing drug block (Lin et al. 2005a; Thurner et al. 2014; Tschirhart and Zhang 2020; Zhang et al. 1999). There is however some complexity to this relationship since quite different results were seen when the extracellular solution was acidified using sodium acetate rather than hydrochloric acid. In this case, while lowered pH still reduced block by quinidine and azimilide, the potency of dofetilide was increased (Dong et al. 2004), with the authors suggesting this perhaps occurred because sodium acetate reduced the intracellular (as well as extracellular) pH (Dong et al. 2004). Furthermore, in experiments examining acidification of the intracellular space, while extracellular pH was maintained in the physiological range, dofetilide, flecainide and amiodarone’s block was not diminished when the drugs were applied extracellularly (Du et al. 2011), while for ibogaine, intracellular application of the drug in the presence of intracellular acidification greatly increased the extent of block (Thurner et al. 2014).

### Mechanism of pH effects on hERG block

Despite differences in drug class and chemical structure of compounds that block hERG, a common explanation for the effect of pH on drug potency has emerged, based on how charge on the functional groups of a drug molecule affects their partition coefficients and hence their ability to cross the cell membrane. For example, antimalarial drugs such as quinine and chloroquine are weak bases and can gain or lose protons from their amino groups depending on pH (Warhurst 1986). In their neutral form, these compounds are lipophilic, with a high partition coefficient (logP), and hence are able to cross the membrane to access their intracellular binding site. However, in more acidic environments, these molecules become protonated, more hydrophilic/lipophobic and less membrane permeable, limiting access to their intracellular binding site and reducing the observed degree of block (Warhurst 1986; Warhurst et al. 2003) (Fig. 1b). Consistent with this, it has been seen that a drug’s potency to block hERG increases with lipophilicity, as measured by logP, or basicity, as measured by pKa (Kawai et al. 2011), while several studies of individual compounds also support this mechanism. For example, Zhang et al. calculated that for verapamil, with a pKa around 8.8, 4% of molecules would exist in a neutral form at pH 7.4, compared to 28% at pH 8.4 and 0.4% at pH 6.4, and observed a corresponding reduction in the potency of block as pH was decreased in vitro (Zhang et al. 1999). The authors also demonstrated that block by N-methyl-verapamil, a permanently charged analogue of verapamil, was not sensitive to changes in pH, confirming that the effect on block was specifically due to the charge on the drug molecule (Zhang et al. 1999). Similar explanations have also been posed for other drugs such as flecainide (Du et al. 2011), ibogaine (Thurner et al. 2014), fentanyl (Tschirhart and Zhang 2020) and hydroxychloroquine (TeBay et al. 2021) supporting the case that this is a common mechanism for the effect of pH on a drug’s potency to bock ERG.

For some drug molecules, however, the picture can be more complicated. Dofetilide has multiple functional groups with different pKa values, including two methanesulfonamide groups, with pKa values of 9.0 and 9.6, as well as a nitrogen atom with a pKa of 7, making it a zwitterion (Du et al. 2011). At a pH of 7.4, 2.5 and 0.6% of the methanesulfonamide moieties are charged, compared with 28.5% of amine groups (Du et al. 2011), while at pH 6.3, 0.2% and 0.06% of the methanesulfonamide and 84% of the amine groups would be charged. Thus, the overall effect of acidic pH is a more charged, membrane impermeant molecule that shows reduced block of hERG at lower pH (Du et al. 2011). Other drugs have pKa values outside of the physiological/pathophysiological range but can also exhibit modified potency of hERG block with respect to pH. For example, flecainide, with a pKa of 9.3, exists in 1.2% and 0.1% neutral form at pH 7.4 and 6.3, respectively (a 12-fold difference), so still exhibits significant changes in observed potency between these pH values. Conversely, at the other extreme, amiodarone has a pKa of 5.6 (98% neutral at pH 7.4 and 83% at pH 6.3) and is not sensitive to pH changes in the same range (Du et al. 2011). Finally, for some drugs such as ibogaine, this same mechanism can also result in internal accumulation of a drug molecule, where under low intracellular pH the drug molecule becomes ionised, and hence trapped within the cell, thus increasing the apparent potency of the drug (Fig. 2b) (Thurner et al. 2014).

In addition to the effect of pH via charge on the drug molecule, a further layer of nuance exists in understanding how environmental pH can alter a drug’s potency to block hERG. In a similar manner to extracellular potassium, pH can also affect hERG channel function and hence influences state-specific drug-channel interactions. Specifically, acidosis is known to accelerate hERG deactivation, affecting the occupation of the open state at a given voltage (Anumonwo et al. 1999; Jiang et al. 1999; Jo et al. 1999; Vereecke and Carmeliet 2000) (Fig. 2a). In relation to this, the neutral form of dofetilide has been reported to preferentially bind to the open state of the hERG channel, while the cationic form preferentially binds to the inactivated state (Wang et al. 2016). Using molecular docking simulations, Wang et al. showed that as the channel transitions between open and inactivated states, there is reorientation of the key residues F656 and Y652 that form the drug binding site. Concomitant with this, cationic dofetilide can change confirmation, bringing its benzene rings closer in an event known as π-π stacking, which allows the dofetilide molecule to bind to the channel and stabilise hERG in the inactivated state (Wang et al. 2016). Therefore, overall, a range of factors including the pKa of the compound, the pH of the extracellular versus intracellular environment, passage to the compounds intracellularly accessed binding site and the compound’s state preference all contribute to the pH effect on hERG block in a compound-specific manner. Furthermore, in the physiological/pathophysiological range of pH, significant changes in hERG block, and hence QT prolongation, can occur, making this an important factor for consideration in relation to diLQTS.

## Temperature

### Effect of febrile temperature on hERG block and drug-induced long QT syndrome

Elevated/febrile body temperature, as a result of illness and infection, is known to alter or exacerbate diLQTS phenotypes in patients. Perhaps, most commonly, this occurs in association with the use of antibiotics such as vancomycin and gentamicin (Varriale and Ramaprasad 1995), or antifungals such as posaconazole (Panos et al. 2016), to treat infection. However, febrile temperatures are also associated with other pathophysiological conditions such as hypertension and diabetes mellitus in patients who may also be prescribed drugs with potential to prolong the QT interval such as enalapril and glyburide, respectively (Varriale and Ramaprasad 1995). In vitro studies that are specific to febrile versus physiological temperature are limited, with inconsistent reports across different drugs. Erythromycin, for example, has been shown to be a more potent hERG blocker at physiological (37 °C) as opposed to ambient (22 °C) temperature, with further increased potency observed at febrile temperatures (42 °C) (Guo et al. 2005). In contrast, for moxifloxacin, no significant change in potency was observed between physiological temperature and 42 °C (Alexandrou et al. 2006). Similarly, our investigations showed that febrile temperature significantly increased the potency of azithromycin as compared to physiological temperatures, while for chloroquine and hydroxychloroquine, potency was significantly reduced (TeBay et al. 2021). Further insights into the effect of temperature on hERG block can be gleaned from experiments performed at subfebrile temperatures, which are far more common in the literature. Lacerda et al. reported that physiological temperatures (35 °C) evoked only a slight change in potency for terfenadine and loratadine (increase or decrease respectively), with no significant changes observed for cisapride and erythromycin when compared to ambient temperature (Lacerda et al. 2001). Contrary to this, other studies report significant effects of temperature on block of hERG by erythromycin (~ sevenfold increase in potency) (Kirsch et al. 2004) — a difference perhaps is a result of the different voltage protocols used between the two studies. In relation to this, Kirsch noted that at 22 °C, erythromycin did not reach steady state of block when employing a 2-s step pulse protocol with a 10-s interval, leading to an inaccurate estimate of IC_50_, while at physiological temperature, the true steady state was reached, because of the faster onset of block. This raises an important point that is equally applicable to any studies assessing hERG potency — that there is no “gold standard” protocol and the observed degree of block can be protocol specific. As a result, this potentially confounding factor should be considered in any comparison between studies, such as those described in this review. Overall, then it is clear from the literature that the effect of temperature on potency is compound specific, meaning consideration of the proarrhythmic risk associated with administration of potentially QT prolonging drugs to patients with fever needs to be made on a drug-by-drug basis.

### How does temperature modify potency of block?

In a similar manner to kalaemic variation, experiments examining the temperature dependence of the potency of hERG block have suggested two potential mechanisms to explain temperature sensitivity: first, through modification of hERG channel function, particularly in relation to binding of state-dependent drugs, and, second, through direct effect on drug interaction with its binding site on the channel protein. In relation to the first of these, hERG electrophysiology displays complex temperature dependence, with increasing temperature causing a negative shift in the voltage dependence of activation, in concert with a positive shift in the voltage dependence of inactivation (Vandenberg et al. 2006), resulting in an overall increased occupancy of the open state at physiological voltages (Fig. 2a). For compounds that exhibit state-dependent binding, these temperature-dependent shifts in state occupancy therefore have potential to affect the measured potency of block. In this regard, Yao et al. investigated the effects of temperature on hERG block by probing state-dependent inhibition with various voltage protocols and temperatures. For astemizole, overall decreased potency was observed at higher temperature, with the greatest degree of block observed with a non-state selective protocol, suggesting that astemizole is able to block multiple states of hERG (Yao et al. 2005). Both terfenadine and ketoconazole similarly showed little preference between protocols optimised for close- or open-state occupancy and, consistent with that, showed little change in potency at higher temperatures. Finally, while E-4031 exhibited open-state preference during ambient temperature recordings, no change in potency was observed at higher temperatures (Yao et al. 2005). The relationship between channel state occupancy, temperature and channel block is therefore complex and requires further experiments across a wider selection of compounds to fully resolve.

The second possible explanation for the effect of temperature on hERG potency — a direct impact of temperature on drug binding kinetics — has been probed using combinations of fast perfusion systems, voltage protocols and in silico modelling. Using ultra-fast solution exchange systems, Windley et al. were able to directly measure both the onset of block and washout of cisapride, showing that the kinetics of both drug binding and dissociation were temperature sensitive and that complex characteristics of kinetics at higher temperatures could be explained by an accumulation of drug in an intermediate, non-blocking state (termed an encounter complex). Furthermore, they showed that in the context of the cardiac action potential, these temperature-dependent effects on drug binding kinetics were important in predicting the degree of prolongation associated with hERG block (Windley et al. 2016). Following this, a study of a broader range of drugs including verapamil, cisapride, bepridil and terfenadine found that while increasing temperature accelerated the observed onset of block (τ_on_) for all drugs, the temperature dependence of association and dissociation rates was compound specific (Windley et al. 2018). Furthermore, while there was no significant effect of temperature on measured potency in steady-state block assays, the alterations to the kinetic parameters alone still resulted in variable temperature dependence of the predicted degree of action potential prolongation for each of the drugs (Windley et al. 2018). Overall, this data therefore supports the need to consider the influence of temperature on the kinetics of drug block, even in the absence of changes to potency, in relation to diLQTS. Furthermore, since the effects of temperature appear to be compound specific, pharmacological screening data for use for risk prediction in diLQTS should where possible be acquired at physiological temperatures.

## Drug coadministration

While most in vitro studies focus on the effect of a single environmental factor on hERG potency, the reality in relation to QT prolongation in the clinical setting is more complex. Patients are often administered multiple drugs with potential to prolong repolarisation, in the background of combinations of electrolyte disturbances and/or chronic disease states (Ayad et al. 2010; Digby et al. 2010). For example, in a study by Digby et al., subjects were prescribed on average 2.8 QT prolonging drugs in the background of diseases including hypertension and dilated cardiomyopathy (Digby et al. 2011). Similarly, in a study of 48 patients hospitalised for TdP, the mean medication number per patient, including QT prolonging drugs in some instances, was 1.1, with electrolyte imbalances seen in 79% of patients (Lazzerini et al. 2018). This data therefore highlights the importance of considering how drugs might interact with each other, either directly or indirectly in understanding QT prolongation in patients.

Regarding direct drug effects, the simplest consideration is that of an additive effect on hERG block. Most drugs that block hERG are thought to share a common binding site formed by a network of aromatic residues in the vestibule of the channel (Kamiya et al. 2008; Stansfeld et al. 2006). Given this common binding site, a patient taking multiple QT prolonging agents could simply be considered to have an increased load of hERG channel block — so increasing their potential for QT prolongation and TdP. In patients, these additive effects have most often been reported in association with coadministration of antipsychotic drugs. Lin et al. reported a patient presenting with schizophrenia who was prescribed risperidone, amisulpride and haloperidol, leading to sudden cardiac arrest, where discontinuation of amisulpride leads to a gradually normalised QTc interval (Lin et al. 2009). In the same study, the authors also described a second patient who developed a QTc interval of 510 ms when co-administered amisulpride and flupenthixol, with neither agent alone producing concerning QT prolongation (Lin et al. 2009).

Aside from additive effects on hERG block, coadministration of drugs can also result in increased torsadogenicity via effects on drug metabolism. Increasing concentrations of berberine or clarithromycin have been shown to significantly inhibit activity of cytochrome P450 enzymes of the CYP3A family in vitro. Since this enzyme is a major metaboliser of many QT prolonging drugs, this reduction in CYP3A activity can lead to altered pharmacokinetics and hence a greater plasma concentration of either drug (Zhi et al. 2015). This link between inhibition of drug metabolism and proarrhythmia has been observed across multiple studies including reports that ketoconazole, erythromycin, diltiazem, itraconazole and grapefruit juice — all inhibitors of cytochrome P450 enzymes — have resulted in increased serum concentration of terfenadine, halofantrine and cisapride, leading to QT prolongation and TdP (Charbit et al. 2002; Paris et al. 1994; Pohjola-Sintonen et al. 1993; Rajput et al. 2010; Thomas et al. 1998). This phenomenon has also been detected in larger cohorts where coadministration of ketoconazole with domperidone was found to triple the plasma concentration of domperidone, exacerbating QTc prolongation to clinically significant levels, over and above that observed for either agent alone (Boyce et al. 2012).

Systemic effects induced by other drugs have also been seen to modify the risk profile of QT prolonging compounds. For example, Roden et al. described cases where hypokalaemia caused by potassium-depleting diuretics were found to exacerbate quinidine-induced QT prolongation (Roden et al. 1986), while incidences of hypomagnesemia caused by protein pump inhibitor usage, in combination with QT prolonging medications such as ceftriaxone or disopyramide, were shown to trigger TdP (Lazzerini et al. 2018). Finally, another case described a patient treated with prednisolone for myasthenia gravis precipitating atrial fibrillation, which was in turn treated with disopyramide. The disopyramide administration resulted in worsening myasthenia gravis, leading to respiratory failure and serum disturbances including alkalosis and hypokalaemia, which together precipitated TdP (Hirose et al. 2008). Together, these cases demonstrate that regardless of the mechanism of their interaction, the simultaneous presence of multiple hERG blocking agents, and their interaction with systemic factors such as electrolytes, have clear potential to increase proarrhythmic risk, and patients should be monitored appropriately when QT prolonging medicines are co-administered.

## Conclusions

In order to understand or predict the occurrence of drug-induced QT prolongation and TdP in patients, it is clear that risk allocation is far more complicated than a static label assigned to individual drugs. Rather, a range of pathophysiological factors associated with disease states as well as coadministration with other drugs need to be considered when prescribing and managing the risk of therapeutics with potential to prolong the QT interval. While significant literature exists describing how factors such as pH, fever and kalaemic variation affect potency to block hERG, there are still gaps in our knowledge regarding the mechanisms of these effects, which may be better addressed via studies on more extensive drug libraries that are now feasible as a result of the increased use of high-throughput automated patch-clamp screening platforms. Furthermore, incorporation of data from these large-scale screens into population models of cardiac electrophysiology (TeBay et al. 2021; Varshneya et al. 2021) will help us better understand the relationships between a drug’s ion channel blocking potency, the effect of environmental modifiers, genetic background and risk of TdP.
